# The Care Dependency Grant for children with disabilities in South Africa: perspectives from implementation officials

**DOI:** 10.1080/0376835X.2021.1981250

**Published:** 2021-10-15

**Authors:** Zara Trafford, Leslie Swartz

**Affiliations:** a Department of Psychology, Stellenbosch University, Stellenbosch, South Africa

**Keywords:** social protection, disability, children, South Africa

## Abstract

For people with disabilities, appropriate social protection interventions can contribute to breaking the cyclical relationship between poverty and disability and may improve social inclusion. In South Africa, a national social assistance programme provides ‘social grants’ to individuals on the basis of poverty, age, or disability. These grants have been extensively studied but there has been little investigation into the Care Dependency Grant, designed to support the care of children with disabilities. These children consistently have far poorer outcomes on key metrics for wellbeing, health, and education than their non-disabled peers. More attention ought to be focused on uplifting this profoundly marginalised population. We present initial findings from interviews with officials at the South African Social Security Agency, the country’s grants implementation agency. These narratives add weight to the growing local and international consensus that complementary interventions and effective intersectoral collaboration may greatly enhance the impact of cash transfers.

## Introduction

There is an urgent need for more attention to be focused on the care and wellbeing of children with disabilities^1^ in South Africa. People with disabilities are among the most vulnerable in any population, partly due to the documented higher direct and indirect costs associated with disability, and the proven cyclical relationship between multidimensional poverty and disability (Hanass-Hancock and McKenzie, 2017; Mitra *et al*., 2017). For disabled children, these vulnerabilities may be intensified by the relative lack of power and autonomy associated with childhood (Banks *et al*., 2017). In LMICs especially, strong social protection programmes for disabled people can improve their quality of life and potentially, facilitate their increased access and social participation throughout the life course (Walsham *et al*., 2019). In South Africa, a relatively progressive national social assistance programme distributes grants (unconditional cash transfers) to large portions of the population on the basis of needs related to poverty, age, and disability. However, a growing evidence base indicates that this form of social assistance may be more effective when it is part of a network of broader complementary interventions delivered by departments collaborating across sectors (Patel, Hochfeld and Moodley, 2013; Saran, White and Kuper, 2020).

Despite South Africa’s strong commitment to disability rights-informed policy, disabled children consistently have far less access to health and educational services than their non-disabled peers and experience high levels of stigma and social exclusion (Philpott, 2014; Shung-King *et al*., 2019). The primary form of social protection available to caregivers of children with disabilities is the Care Dependency Grant (CDG). Extensive research has shown the positive impact of South Africa’s social assistance programme, with particularly large literatures on the adult disability grant (DG) and the child support grant (CSG) (Knight, Hosegood and Timæus, 2013; Patel, Knijn and Van Wel, 2015; Zembe-Mkabile *et al*., 2015; Godfrey *et al*., 2016). In contrast, although the impact of the CDG is explored in the early childhood development literature (Philpott, 2014; Elphick, De Sas Kropiwnicki and Elphick, 2015; Moodley, 2021), only a handful of studies have the CDG as their main focus (Berry and Smit, 2011; Martin, Proudlock and Berry, 2014; Letsie, 2016). In addition, due to major gaps in the evidence base, neither the prevalence of childhood disability nor the uptake rate of the CDG can be properly measured (Budlender, 2019). These factors make it difficult to advocate for and monitor change for children with disabilities.

As part of a broader study to gather multiple stakeholder perspectives on the CDG, this paper presents findings from a series of interviews with officials at the South African Social Security Agency (SASSA). SASSA administers and distributes all of the social grants in the country. SASSA reports to the national Department of Social Development (DSD) but is not provided with guidance on day-to-day operations (Martin, Proudlock and Berry, 2014; Kelly, 2016a). SASSA has thus instituted its own regulations and processes for grant applications and assessments, as informed by eligibility rules in the Social Assistance Act (SSA) of 2004 (South African Presidency, 2004). To approve or deny applications, a medical assessment performed by a doctor is combined with an income threshold assessment or ‘means test’ (Kidd et al., 2018). In light of the higher costs associated with disability, the CDG amount is around four times the size of the CSG (for non-disabled children) and the income threshold for application is also around four times higher. Practically though, the vast majority of people who are likely to access any kind of grant (and indeed, the overall population) generally live far below the income CDG income threshold (Shung-King *et al*., 2019).

Insights from people who are involved in the actual implementation of bureaucratic systems can produce a better understanding of how these kinds of programmes works in practice, rather than on paper (Heyman, 2012). SASSA officials’ narratives make a needed contribution to the minimal evidence base around the CDG, as well as highlighting some challenges and opportunities for improvement. Despite the scale and intensity of the barriers facing children with disabilities and the potential of social assistance to moderate some of these barriers, incommensurate attention has been paid to the CDG. Without the inclusion of these children and their families, we cannot make progress toward the country’s disability-rights and broader developmental goals (SAHRC, 2017).

## Methods

The Stellenbosch University Research Ethics Committee: Social Behavioural and Education Research (REC:SBE13097) approved this research. Permission to interview SASSA staff was obtained from the national Department of Social Development. A longer list of possible participants was provided to the first author, after personal introduction to a senior official. The first author approached potential participants directly, to minimise any sense of professional obligation to participate. The voluntary nature of participation was noted during recruitment, consent, and the interview. Data in this paper is from six in-depth, semi-structured interviews with five participants at various levels of the organisational hierarchy, including administrators and management of different genders and races. Interviews were conducted between July 2020 and April 2021. Participants were anonymised using numbers assigned according to the order of interviews (e.g. P1 denotes the first participant).

Informed consent forms were available in Afrikaans, English, and isiXhosa. Verbal and written consent were obtained. Participants confirmed they were comfortable being interviewed in English, and all are required to use English in professional duties. Due to COVID-19, interviews were conducted remotely using video-conferencing software. Interviews were 1-1.5 hours long. All interviews were conducted and transcribed verbatim by the first author. Atlas.ti facilitated a thematic analysis. For this paper, we selected themes including SASSA staff perceptions of: the intention of the CDG, its actual use, and the challenges and opportunities involved in implementing this grant. This paper reports on only this sample but is the first of a series of forthcoming publications, which will incorporate varied stakeholder perspectives. A strength is that there is currently no other empirical research focused specifically on SASSA officials’ perspectives on the CDG. The participants were at different hierarchical levels but their perspectives were consistent with one another, and interviews conducted with other stakeholders have produced similar themes.

## Results

### Narratives on the purpose of the CDG

As with other unconditional cash transfers, the use of the CDG is not formally dictated by government. When asked what the intention of the CDG was, participants drew on eligibility guidelines in the Social Assistance Act. Officials explained that the primary consideration ought to be the degree to which a child is permanently reliant on care, as inferred from a medical assessment of their functional capacities.

…[we intend to] give grants to those that meet the eligibility criteria… when it comes to a care dependency grant… [the assessor should decide] whether… this child…warrant[s] permanent care … [because] they can’t operate alone (P2) [the CDG gives] assistance to children … if they’re mental[ly]… or physical[ly] disabled, to assist them in their needs… [because the child] cannot care for himself (P4)

When asked how people might be spending the CDG respondents focused on the human resources required to provide consistent care to the child. They also listed a variety of other costs associated with childhood disability, but stressed that using the grant for these costs was not technically part of the official intention of the grant.

…that parent obviously cannot work because that child needs 24/7 assistance (P4) …[maybe a] neighbour comes when [the caregiver] needs to go to work [and the caregiver] needs to give them something [in exchange]. So, it’s around the care provisioning of the child… (P2)

[The CDG might] could be used to pay… a nanny, [in contrast to] a child who doesn’t have special needs [and] doesn’t require the 24/7 attention. Often a child with special needs needs additional medication… Or assistive devices… [and] children with special needs sometimes need nappies until they are… far beyond the potty-training stage (P1)

Officials specifically emphasised, however, that the grant is not actually intended to cover all of the costs associated with providing for a child with disability, and that the burden of these costs was not supposed to be incorporated into eligibility considerations. Rather, the higher means test threshold and larger grant amount (relative to the CSG) had been instituted to partly offset additional costs.

### Perceptions of CDG benficiaries’ actual use of the CDG

There is currently no system for monitoring caregivers’ use of any of the grants, including the CDG. The SASSA participants we interviewed believed that as long as the recipient was eligible and had been assessed properly according to SASSA guidelines, the money was used ‘appropriately’ (i.e. primarily for the child with high care needs). However, SASSA officials also explained that in the context of high levels of poverty and inaccessibility of services, it was likely that the CDG might actually be being used more flexibly to support the wellbeing and survival of the broader household. Officials were not overly critical of such recipients, but did emphasise that this was not the official intention of the grant.

When asked if they thought that recipients were misusing the CDG, SASSA representatives gave differing estimates based on their own observations. With the exception of one, SASSA representatives felt that caregivers who misused the CDG were in the minority.

…for the most part… it is being used… for the purpose that it is there. … you will always find sections of the population…that misuse… but I really think that [with the CDG], it’s a very small percentage … maybe 5% (P1)

…these are cash transfers [so] there’ll always be caregivers, maybe 20%, who misuse it… my understanding is that those would be in the minority (P2)

In contrast, one participant who worked in financial administration and lived in an area where there were many CDG recipients, felt that there were more *un*deserving (‘devil’) than deserving (‘angel’) beneficiaries of this grant and was distressed by its misuse.

…[supposedly, the] child cannot even stand for ten minutes – but that child is 24/7… playing in the streets! … I see more devils than I see angels. … I’m not talking about what is happening [statistically]…I’m talking about…what I actually see [in my neighbourhood] … when it’s care dependency grant’s pay-out… the father will… not even go to work [and] you will see he will be drunk … If the child wants something… then they buy [the child] an expensive pair of [sneakers]… How do you improve that child’s life [that way]? (P4)

This respondent also had a disability and had previously been an adult DG beneficiary, but had found training opportunities and moved into formal employment. They were angry with the lack of motivation they saw in their community, but situated responsibility for this situation partly on the government. They felt that broader efforts at development and upliftment were severely lacking and that most people who had grown up in poorer environments had not had adequate education or opportunities, which contributed to their misuse of funds.

Before [a grant recipient] even gets money, it is at a loan shark [because they are in debt]. … The thing is… [people living in poverty and in under-resourced areas] won’t know about [educational and empowerment initiatives]. And they won’t tell the children … We (i.e. people living in poorer communities) only know about grants … We (i.e. the government and its implementing agencies) pay our grants, but we don’t look after our people (P4)

Their strong feelings about the misuse of grants were thus balanced with an understanding that a cash transfer alone cannot moderate the deeply-rooted effects of under-development. These sentiments were echoed by another senior official who noted that while broader developmental work was critical, it had been severely constrained by a lack of resources and difficulties with coherence between different Departments.

### 3 Challenges and successes in the implementation of the CDG

#### Limits to the legislation and confusion about the purpose of the CDG

According to the Social Assistance Act, eligibility for a CDG ought actually to be assessed according to whether a child needs ‘permanent care *and/or support services ‘*. However, since SASSA does not have the autonomy to alter eligibility definitions and ‘support services’ have not been clearly defined, consideration of the child’s need for support services is generally disregarded, in practice.

…‘support services’ has… not been clearly defined [in the legislation] … [that’s] where the question marks come in (P1)

…as policy implementers… we wouldn’t know how to… put parameters around it…SASSA… can’t… [just] define support services [by imposing our own understandings] (P2)

Currently, medical assessors must gauge a child’s need for care based on the doctor’s *clinical* understanding of childhood developmental milestones and associated functional capacities and incapacities. However, partly because this is a non-contributory scheme, SASSA cannot mandate the use of resource-intensive standardised tests. SASSA officials described how this unintentionally leads to more subjectivity in disability-related grants assessments and in turn, produce highly variable outcomes. As non-clinicians, they also felt ill-equipped to properly guide clinical assessors.

…we cannot… as non-clinical people, guide… [what] impairment would equate a disability… that’s… also part of the problem, with doctors struggling to make [standardised] assessments… (P1)

We rely… on the… perspective of a clinician, against them understanding… the law. … a clinician *might* use standardised tests to strengthen their argument, but we [can’t formally require these], because of the [resource] deficiencies we have… (P2)

SASSA staff were at pains to clarify that according to the existing law, the presence of a formal diagnosis of disability does not automatically equate to eligibility for the CDG. Participants indicated that when assessors have (accidentally or purposely) misunderstood the purpose of the CDG, grants have been awarded on the basis of a diagnosis and are then approved grants for anyone in this community with the same diagnosis. Participants were aware of the limitations in the current system and expressed sympathy for clients and medical assessors. However, they were also frustrated by the fact that since assessments were so variable, it was difficult to standardise community understandings of the CDG and ensure that it was awarded on the same basis in different places.

A child with diabetes…might not meet the law, as it’s [currently] crafted. Child goes to school in the morning, they are given the insulin, coming back they are given the insulin. Can you see that child is not 24-hour dependent? But… in a particular area, all those children were given the [CDG] at the age of two [so there is now an expectation that a diabetes diagnosis entails a grant]. [Simlarly,] you find in the past, all children with dyslexia… without a comorbid[ity]… were given grants. That’s not the intention of what the law was. …[But] society and politicians… want to take us to a diagnostic level. … They think someone with autism qualifies for a grant. They think someone with albinism qualifies for a grant. It *is* even complicated. Because…doesn’t the World Health Organization say, ‘albinism is a disability’? [But] not from the perspective of social assistance [legislation] (P2)

Finally, SASSA officials suggested that desperate people might still try to access disability-related grants even when they understand their actual purpose. Those living in areas with higher beneficiary numbers are also likely to be aware of the variability of assessment outcomes and may be willing to go through multiple assessments in the hope of finding a sympathetic assessor, which adds to already heavy systemic pressures.

…people come and flood the system because they don’t understand… what is the intention of disability-related grants. [Sometimes] because they are facing poverty, people want to test the scope and say, ‘I have a condition thus I want a grant’ (P2)

#### Difficulties relating to the ‘permanence’ of the CDG

Adult DGs can be awarded on a 6-month or 1-year basis, and applicants must then undergo ongoing medical assessments to continue testing for eligibility. To date, the CDG has only been available as a ‘permanent’ grant, awarded monthly from the time it is approved until the child turns 18. However, as reported above, CDGs have previously been awarded to the caregivers of children with chronic (but treatable or curable) illness, or with long-term (but not necessarily permanent) higher than usual care needs. In these instances, the CDG might have been disbursed long after the child’s condition had been resolved. In the broader context of the grants programme, there are profound concerns about awarding any long-term grants, due to the overall cost of the national programme and the preponderance of fraud.^2^ Officials felt that the lack of a shorter term option for the CDG may have led to both ‘wastage’ on those who no longer needed support and at the same time, the potential exclusion of those who really did need permanent support.

…doctors are really wary about giving permanent grants, [so] you will have them classify a permanent condition as temporary… [because they think,] ‘if I give it as a temporary grant the person’s got to come back and if [SASSA] loses money, we’re not going to lose too much’… (P5)

… [say] a child presents with a heart defect at age…1, 2 years old… [with] medical intervention the child might, by age 5, not be care dependent. … [assessors] find that very difficult… because the assessment form and the Act currently say that, if you get a care dependency grant, you get it until you are the age of 18 (P1)

An additional challenge highlighted by SASSA staff was that the transition from the CDG to the adult DG was too slow or sometimes did not happen, even when an applicant clearly met relevant eligibility requirements. Respondents felt that this was mostly rooted in the fact that too many inclusion errors in the existing CDG database prevented automatic transitions to the DG.

…we are not at a stage where we would …[automatically] migrate them [to the adult DG] … because of the inclusion errors that we find in our [CDG] dataset (P2)

#### Successes and opportunities

Current successes and opportunities for progress included an upcoming policy adjustment and a recent shift toward increased electronic integration. A senior respondent indicated that the Social Assistance Act is currently under review and an updated version ought to be promulgated at some point in 2021. The updated regulations make provision for a temporary CDG. However, this will entail the design and implementation of a range of new training and administrative processes, which may be time and resource intensive.

…the new regulations…allow…for a temporary care dependency grant… we [will] need to reorientate our assessing doctors, because they’re used to the fact that a care dependency is for a permanent condition, so it would take the reorientation of doctors [and] our own staff …we would [also] have to bring in a… medical review process for the care dependency grant, which is not in place at the moment (P5)

Interestingly, although the COVID-19 pandemic has caused overwhelming systemic pressure on the grants programme and a breakdown of the disability-related grants application process, it has also expedited SASSA’s movement toward electronic systems management. The latter may, in the longer term, improve the system’s efficiency and efficacy.

…we have developed an electronic… tool, which helps us manage the bookings for clients … we’re [also] launching… an online booking tool… so [clients] can book their own medical assessment… COVID has pushed us to do it a lot quicker … [We are] looking at expanding the tele-med concept … [For children whose medical report indicates a severe, permanent disability, we can] use that medical report [to award the CDG] … Then when the child turns 18, [we can] do the assessment for the [adult] disability grant … [we can then get more information] through a telephone assessment with the caregiver. … we will continue implementing [this] beyond COVID, because it just makes sense (P5)

## Discussion

Approaches to social protection and disability in South Africa are fragmented, and services are commonly delinked from or incoherent with one another (Philpott and Nithi Muthukrishna, 2019). This is partly because the models for social protection in South Africa were initially based on northern welfare systems, developed in the 1950s-1960s and drawn from an individualised conception of need (Patel, 2018). During apartheid, state-sponsored funds were distributed to needy groups within the small white minority at the explicit exclusion of the vast majority of the country’s population (Kelly, 2016a). After the democratic government came into power in the mid-1990s, these models were deracialised and extended to include the whole population. However, the grants landscape was developed piecemeal and was not thoroughly overhauled, despite the need to cater for exponentially more people than originally planned. Decentralised governance and the complexities of service delivery in a large country with highly uneven resource distribution continue to make intersectoral collaboration and national standardisation difficult. Attempts are now being made to move toward an integrated system of ‘comprehensive social protection’, but this vision has not yet been realised.

With regard to children with disabilities specifically, many of the potential uses for the CDG listed by our respondents ought technically to be provided by other government departments and sectors, rather than being paid for by individual caregivers out of their SASSA grant. For example, the Department of Health is supposed to facilitate access to appropriate assistive devices, and the Department of Education is tasked with providing appropriate educational services for children with disabilities (Philpott, 2018; Philpott and Nithi Muthukrishna, 2019). However, as progress toward the proper realisation of children with disabilities’ rights has to date been sluggish and limited (Philpott, 2014; Moodley, 2021) the CDG has functionally become the primary source of government support for the social protection of children with disabilities and their families (Elphick, De Sas Kropiwnicki and Elphick, 2015).

### Reflecting on SASSA perspectives

SASSA respondents appeared to have generally pragmatic and empathetic attitudes toward economically poor caregivers of disabled children. This contrasted with our initial expectation that SASSA staff might have cynical or beleaguered attitudes toward CDG recipients, based on the heavy load and prior literature about the tensions involved in the disability-related grants programme (Knight, Hosegood and Timæus, 2013; Kelly, 2017). All of these SASSA participants had lived or worked in close proximity to poor, underserved communities. Although they did not endorse the practice of sharing funds, they were aware that recipients may have been using at least some of the funds from the CDG to support their whole household. This pattern is also reflected in other local studies regarding the use of social grants, and has been shown to be an important means of survival and avenue through which care is obtained in a household (Gutura and Tanga, 2017; Mtyingizane, 2018; Ketani and Tanga, 2019; Granlund and Hochfeld, 2020). Further, they explicitly did not condone the misuse of grants and were concerned about wastage and fraud, but most did not place the blame on grant recipients alone. Overall, their narratives represented a nuanced perspective on the ways in which difficulties with intersectoral collaboration and the lack of access to important resources or services in poor communities may contribute to the misuse of funds, or attempts to gain access to a grant when individuals are ineligible.

Although it is SASSA’s primary governing document, the Social Assistance Act currently lacks comprehensiveness and specificity with regard to disability (Martin, Proudlock and Berry, 2014; Kelly, 2016b). Limitations in the existing CDG legislation were cited as being a key challenge for the implementation agency. Nonetheless, officials returned to the specific wording of the SSA when faced with questions about the purpose of the grant, the inconsistency of assessments, or insufficiencies in the current system. This return to the wording of the SSA did not seem to be an attempt to defer responsibilities. Instead, it appeared to represent frustrations with a lack of autonomy to make changes to the law, despite being tasked with its implementation and their observations of pitfalls in its practical application.

The perceived subjectivity of CDG assessments was another key administrative challenge. SASSA participants felt that this subjectivity was somewhat unavoidable due to resource constraints, but that it had unfortunately led to variable outcomes and a widespread misunderstanding of the purpose of the CDG and DG. One participant in this study preferred to call these grants ‘disability-related grants’ rather than ‘disability grants’, as he felt that this better represented the purpose of this grant category: to moderate the *effects and implications* of a disability or impairment, not just to be awarded on the basis of a diagnosed disability. In the case of the CDG, assessments are based a child’s adherence to or divergence from standard developmental milestones and as a related consideration, whether the child requires continuous care to perform their daily activities. However, a need for ‘permanent care’ is harder to ascertain than the relatively more binary concept of a formal diagnosis (although as disability operates on a spectrum, even obtaining a formal diagnosis may be difficult). This kind of assessment is also inherently more subjective because conceptions of ‘need’ and ‘care’ are contingent on variables including capability, cultural norms, and resource availability, all of which may be influenced by the doctor, caregiver, and/or child’s individual and environmental factors.

Respondents indicated that confusion about the purpose of the CDG has made it hard to standardise the programme, make sure that it is equitably implemented, and improve the general population’s understanding of the purpose of this grant. A previous qualitative study also highlighted the subjectivity of disability grants assessments and described how assessors drew more heavily on their own interpretations of need and deservingness (influenced by their personal and professional backgrounds), rather than basing their decisions solely on SASSA’s guidelines (Kelly, 2016a, 2016b). SASSA participants expressed concerns that assessors might continue to inappropriately withhold access to or award the CDG on the basis of a diagnosis or their personal opinions, rather than on close adherence to SASSA guidelines. Alternatively, some participants indicated that the preponderance of fraud and misuse in the disability-related grants programme has made medical assessors wary of approving permanent grants, even when they would be most appropriate. These patterns may unintentionally have made it more difficult for those who would meet the CDG eligibility requirements to access this important support, due to hardened attitudes among assessors or backlogs limiting access to assessors.

It is encouraging that progress through policy change is underway. The current lack of distinction between chronic disability and shorter term illnesses may be contributing to inaccessibility for those who need the CDG most (Martin, Proudlock and Berry, 2014), such as children whose disability has not yet been diagnosed or those who have struggled to access care services and referral to other kinds of support. Although this may have been useful for expanding the positive impact of income support for those who do have an urgent (but not permanent) need to provide additional care to their child, it has also been shown to contribute to overloading an assessments system that already struggles to sustain itself (Kelly, 2016a). Shifts in legislation may allow for more accurate targeting and a clearer distinction between temporary CDGs (for shorter term but chronic care needs), and the specific needs of a child with permanent impairments. The creation of an option for shorter term social assistance may also eventually help to minimise the inclusion errors that currently exist in the CDG database, which could smooth the transition to the adult DG for those who need it and decrease negative community opinions toward CDG recipients (Letsie, 2016). The adoption of electronic systems for managing the assessment of these grants is also likely to assist in improving efficiency and may make a rapid and valuable contribution to the evidence base regarding childhood disability types and prevalence in South Africa.

While legislative policy changes are a critical first step toward change, however, they do not automatically result in accurate or uniform application in implementation. It is important to remember that all of these new processes and regulations will take time to translate into implementable guidelines. It will also take time for the perspectives and practices of medical assessors, SASSA officials, and the public at large to shift. For example, the ongoing disregard for half of the current definition of eligibility for the CDG (i.e. ‘…and/or support services’) has been reported on as a gap for almost a decade and is, technically speaking, illegal (Martin, 2014). It is also possible that implementing an additional grant type may introduce additional confusion and add to the administrative burden already represented in the constant cycle of reapplications for temporary adult DGs. Finally, it is unlikely that these kinds of technicist changes will resolve the broader socioeconomic and contextual issues that contribute to misunderstandings about the purpose of the CDG. While the shift toward the availability of a temporary CDG responds to one of the key challenges cited by SASSA participants, it remains to be seen how the relevant administrative arrangements will be made, or whether this will actually have a positive effect for children with disabilities and their families in South Africa.

## Conclusion

Globally, and especially in LMICs, the use of non-contributory cash transfers has been proven to be an important and valuable intervention. However, the current consensus is that cash transfers are most effective as one node in a network of comprehensive, interlocking social protection efforts (Sabates-Wheeler and Devereux, 2008; Molyneux, Jones and Samuels, 2016; Patel, Hochfeld and Chiba, 2019). Similarly, in order to flourish, a disabled child (or adult) ought to be provided with various kinds of support that act together to protect, uplift, and empower (Philpott and N Muthukrishna, 2019). These efforts rely on strong intersectoral collaboration (Britto *et al*., 2014) and in the South African context especially, a cascade down to community levels (Mkabile and Swartz, 2020). Many of these important linkages are currently too weak or non-existent and various ideological commitments to persons with disabilities have not yet translated into the *realisation* of their rights (Martin, Proudlock and Berry, 2014; Martin-Wiesner, 2018).

Many of the factors that detract from the efficacy of the disability-related grants programme are actually related to failures in areas that SASSA does not have the capacity to act upon, such as the provision of strong and equitable health and educational services, adequate transport, and safe housing. There are certainly numerous examples of systemic and other failures at SASSA (Vally, 2016; du Toit, 2017; Gronbach, 2020), but it is also true that no one government department or agency can carry these responsibilities alone. However, as grants have become the primary form of income for so many households, SASSA holds the purse strings on survival and bears a significant proportion of what should be an *intersectoral* responsibility. It is important that we strengthen these crosscutting government departments’ understanding of and commitment to their responsibilities with regard to upholding the rights of disabled children.

The findings in this paper support the argument that the CDG may be perceived as a catch-all for the provision of a wider set of services and systems that ought to be in place to support these children and their families, despite this not being its official intention. It is true that for very poor families, the CDG might make the key difference toward the survival of the whole family unit (Letsie, 2016; Khan *et al*., 2020). However, if additional services and entitlements were available to support the multidimensional needs of these families, the grant would likely have a dramatically enhanced impact (Lloyd-Sherlock and Agrawal, 2014). The CDG might then have far more potential to sustainably and thoroughly move people out of the poverty-disability cycle, which would benefit these children and their families, and would also move the country closer to its developmental and economic goals.

## Data Availability

The data that support the findings of this study are available on reasonable request from the corresponding author (ZT). The data are not publicly available due to the ethical constraints under which they were collected. Transcripts contain identifying information that could compromise the anonymity and privacy of research participants.

## Footnotes

1 Disability terminology is contested. Some prefer “person-first” terminology. Others prefer alternate terminology (e.g. “disabled person”), to emphasise that the oppressiveness of disability results more from constraints in social, political, and geographical environments than from an individual’s impairment. These terms are used interchangeably here.

2 The preoccupation with fraud in the South African political context and grants system will be the central focus of a forthcoming article.
